# A single point mutation expands the applicability of ostreolysin A6 in biomedicine

**DOI:** 10.1038/s41598-023-28949-7

**Published:** 2023-02-07

**Authors:** Anastasija Panevska, Nastja Čegovnik, Klavdija Fortuna, Alen Vukovič, Maja Grundner, Špela Modic, Gregor Bajc, Matej Skočaj, Martina Mravinec Bohte, Lara Larisa Popošek, Primož Žigon, Jaka Razinger, Peter Veranič, Nataša Resnik, Kristina Sepčić

**Affiliations:** 1 Department of Biology, Biotechnical Faculty, University of Ljubljana, Jamnikarjeva 101, 1000 Ljubljana, Slovenia; 2 Agricultural Institute of Slovenia, Hacquetova Ulica 17, 1000 Ljubljana, Slovenia; 3 Institute of Cell Biology, Faculty of Medicine, University of Ljubljana, Vrazov Trg 2, 1000 Ljubljana, Slovenia

**Keywords:** Biochemistry, Lipids, Proteins

## Abstract

An aegerolysin protein ostreolysin A6 (OlyA6) binds to cholesterol-complexed sphingomyelin and can be used for specific labelling of lipid rafts. In addition, OlyA6 interacts with even higher affinity with ceramide phosphoethanolamine (CPE), a sphingolipid that dominates in invertebrate cell membranes. In the presence of pleurotolysin B, a protein bearing the membrane-attack complex/perforin domain, OlyA6 forms pores in insect midgut cell membranes and acts as a potent bioinsecticide. It has been shown that a point mutation of glutamate 69 to alanine (E69A) allows OlyA6 to bind to cholesterol-free sphingomyelin. Using artificial lipid membranes and mammalian MDCK cells, we show that this mutation significantly enhances the interaction of OlyA6 with sphingomyelin and CPE, and allows recognition of these sphingolipids even in the absence of cholesterol. Our results suggest that OlyA6 mutant E69A could serve as complementary tool to detect and study cholesterol-associated and free sphingomyelin or CPE in membranes. However, the mutation does not improve the membrane-permeabilizing activity after addition of pleurotolysin B, which was confirmed in toxicity tests on insect and mammalian cell lines, and on Colorado potato beetle larvae.

## Introduction

Ostreolysin A6 (OlyA6; UniProt: P83467.2, GeneBank: AGH25589.1; PDB: 6MYI) is a 15-kDa protein produced by the edible mushroom *Pleurotus ostreatus* (oyster mushroom). It belongs to the larger protein family of aegerolysins (Pfam 06355; InterPro IPR009413), and has recently gained significant interest due to its ability to bind organism-specific membrane sphingolipids^1–6^. In addition, in the presence of the 59-kDa protein partner pleurotolysin B (PlyB), which bears a membrane-attack complex/perforin (MACPF) domain and is also produced by *P. ostreatus*, both OlyA6 and PlyB can assemble into larger pore-forming complexes in target membranes that contain the aegerolysin lipid receptor^1,4,7,8^. This pore formation results in direct cell death, or in the creation of a passageway for other molecules that can kill the cell.

In particular, OlyA6 can specifically sense (*k*_*D*_, ~ 1 μM) the combination of the two most abundant and important raft-residing lipids, cholesterol and sphingomyelin^1,2,9^. Thus, recombinant OlyA6 variants fused with fluorescent proteins can be used as markers for these important membrane domains. These OlyA6-based markers have relatively small molecular weights, are not cytotoxic, have optimal fluorescence properties, are stable and do not oligomerize in the membrane^2^. OlyA6-based lipid raft probes have been demonstrated to be useful for studies of lipid raft structure and function in fixed and living mammalian cells^2^, and as complementary tools to investigate sphingomyelin-sequestered cholesterol membrane pools^9^, and cellular cholesterol transport^10^. Furthermore, OlyA6 can interact with high affinity (*k*_*D*_, ~ 1 nM) with artificial lipid vesicles and biological membranes that contain physiologically relevant concentrations (1–5 mol%) of ceramide phosphoethanolamine (CPE)^4,8,11,12^, which is a sphingolipid that dominates in invertebrate (particularly insect) cell membranes^13^. Through this CPE binding, OlyA6/PlyB cytolytic complexes have been shown to act as potent and species-specific bioinsecticides, for use against selected coleopteran pests, such as the Colorado potato beetle (CPB) and the western corn rootworm^4^. Recently, a mollusk- and cnidarian-specific CPE analogue, ceramide aminoethylphosphonate, was also recognized as a high-affinity receptor for OlyA6 and a target for cytolytic OlyA6/PlyB complexes^5^, suggesting the use of these complexes for selective elimination of organisms that harbor this sphingolipid in their membranes (e.g. marine invertebrates and oomycetes).

During an extensive investigation of lipid-binding properties of a series of OlyA6 mutants, Endapally et al.^9^ demonstrated that the specificity of OlyA6 to cholesterol-complexed sphingomyelin is driven by a single glutamic acid at position 69. The mutation of this amino acid residue to alanine (E69A) abolished this specificity, whereby the mutant (PDB: 6MYK) also bound to cholesterol-free sphingomyelin. Importantly, the binding of the OlyA6 mutant E69A to sphingomyelin-containing membranes is 100-fold that of the OlyA6 wild-type protein^9^. This important finding has triggered us to further explore the potential applications of OlyA6 E69A (henceforth: E69A), and its fluorescent variant, E69A-mCherry, in biomedicine and agriculture.

In this work, we have assessed the binding of E69A, and the membrane-permeabilizing activity of E69A/PlyB on artificial membranes containing physiologically relevant amounts of the high-affinity aegerolysin receptor, the CPE, both in the presence of cholesterol or without it. Further, the ability of E69A-mCherry to bind free and cholesterol-sequestered sphingomyelin membrane pools, alone and in combination with the EGFP-tagged wild type OlyA6, was studied using the mammalian MDCK cells. Finally, the insecticidal potential of E69A was assessed against Sf9 insect cells, and in toxicity assays against CPB larvae.

## Results

### E69A interaction with sphingolipid-containing model lipid membranes

#### Binding of E69A to sphingolipid-containing artificial membranes

**Figure 1:**
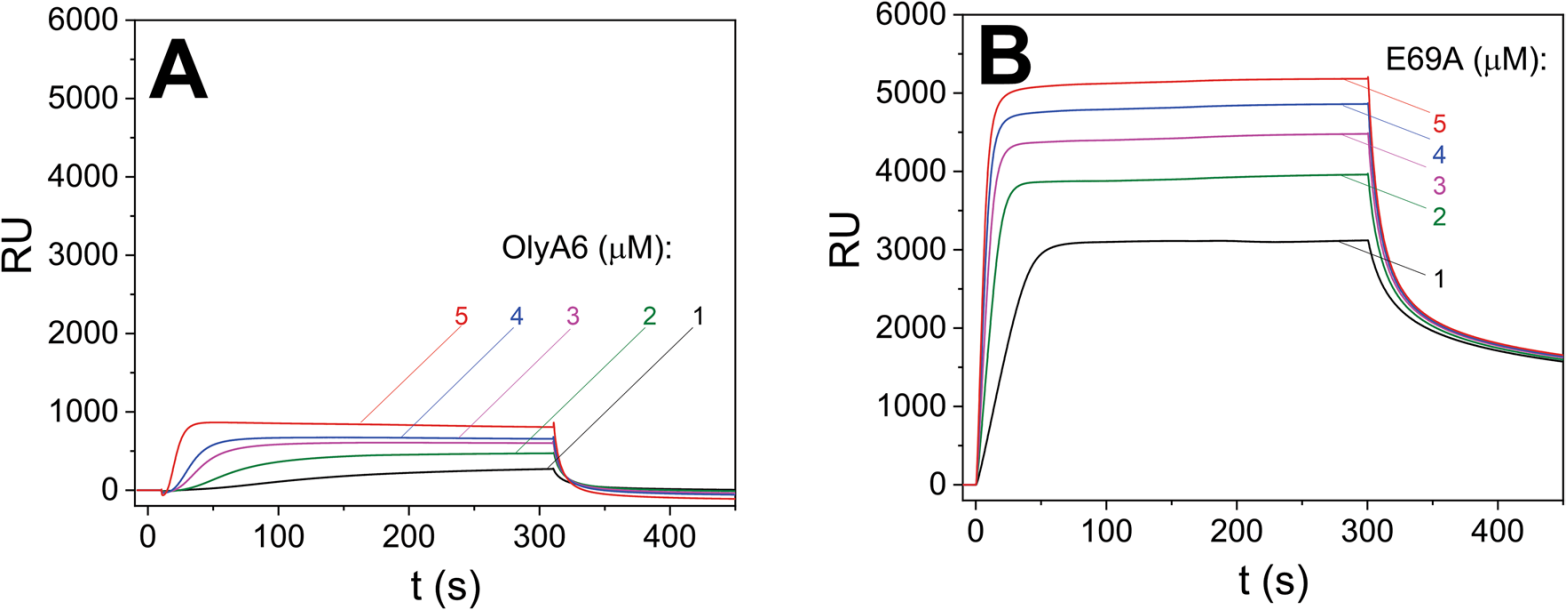
Surface plasmon resonance of the interactions of OlyA6 (**A**) and E69A (**B**) with large unilamellar lipid vesicles composed of total lipids extracted from the Sf9 cells. The vesicles were immobilized (Biacore L1 chip) to approximately 7000 RU and the proteins were injected in running buffer (flow rate, 10 μL/min), using the kinetic titration approach in a multi-cycle by injections of 1–5 µM concentration. Representative sensorgrams of triplicate analyses are shown.

Multi-cycle kinetic experiments with chip-immobilized equimolar LUVs (SM/POPC, SM/Chol, CPE/POPC, CPE/POPC/Chol) show that OlyA6 (0.25–1 μM) binds specifically to SM or CPE only in the presence of cholesterol (Figs. S2A,B and S3A,B). In contrast, E69A (0.25–1 μM) can interact with these LUVs with or without the presence of cholesterol, and its interaction with cholesterol-supplemented LUVs is much stronger than in the case of OlyA6 (Fig. S2C,D and S3C,D). Moreover, E69A binds even fivefold more strongly and stably than the wild type protein, OlyA6, to LUVs obtained from the whole lipid extract of insect Sf9 cells (Fig. 1).

**Figure 2:**
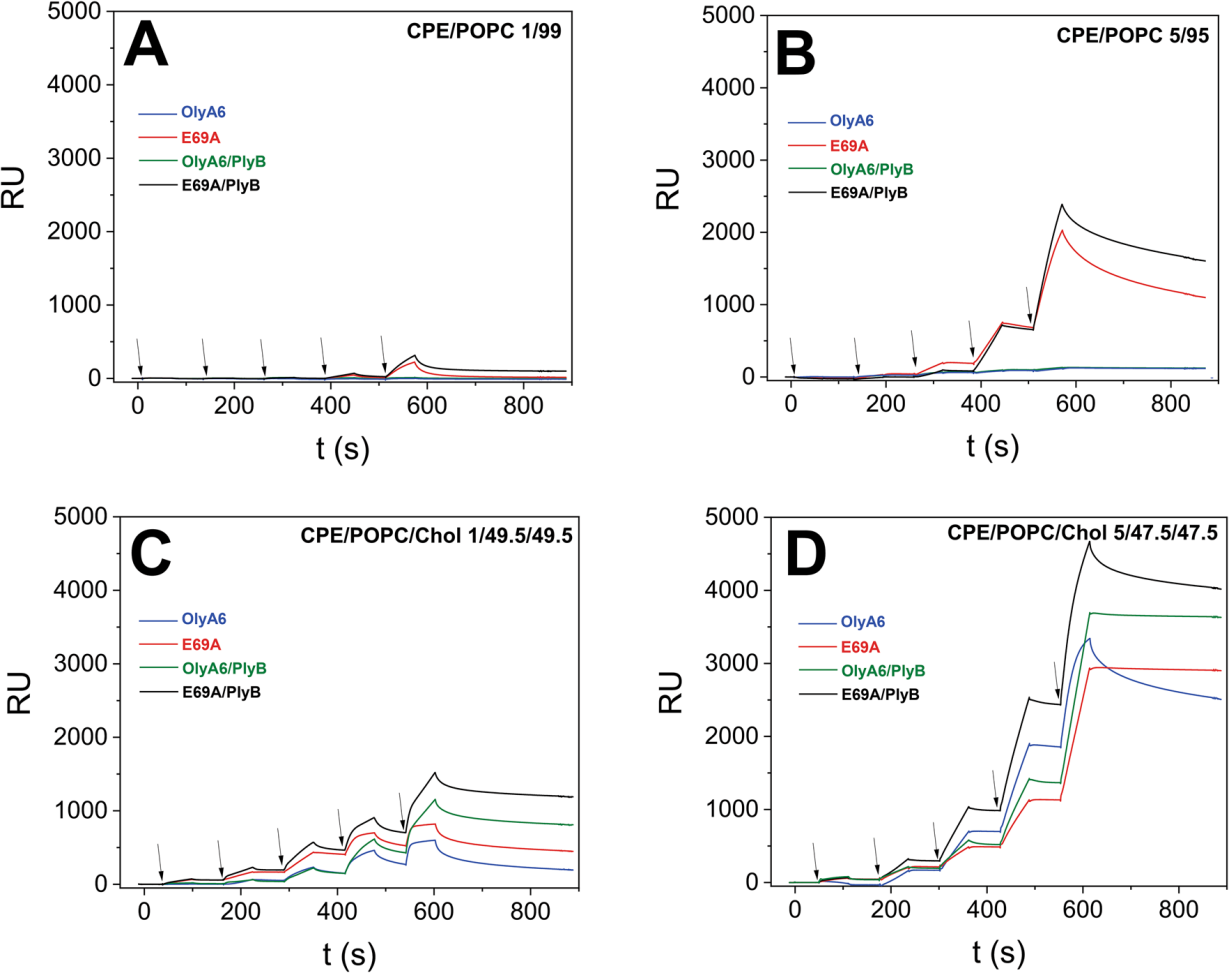
Surface plasmon resonance of the interactions of OlyA6 and E69A, alone or in combination with PlyB, with large unilamellar lipid vesicles composed of various molar proportions of CPE, POPC and cholesterol. The vesicles were immobilized (Biacore L1 chip) to approximately 8000 RU and the proteins were injected in running buffer (flow rate, 10 μL/min), using the kinetic titration approach in a single-cycle by successive injections of 0.03, 0.06, 0.12, 0.25, and 0.5 µM (from left to right, indicated by arrows) concentration. Representative sensorgrams of triplicate analyses are shown. (**A**,**B**) Binding of OlyA6 or E69A, alone or in combination with PlyB, to cholesterol-free lipid vesicles containing 1 (**A**), or 5 mol% CPE (**B**). (**C**,**D**) Binding of OlyA6 or E69A, alone or in combination with PlyB, to cholesterol-containing lipid vesicles supplemented with 1 (**C**), or 5 mol% CPE (**D**). Aegerolysin/PlyB molar ratio = 12.5/1. Lipid molar proportions are indicated on the graphs. *CPE* ceramide phosphoethanolamine, *POPC* 1-palmitoyl-2-oleoyl-*sn*-glycero-3-phosphocholine, *Chol* cholesterol.

Single-cycle kinetic experiments using on-chip immobilized LUVs composed of POPC supplemented with low molar proportions (1 or 5 mol%) of the high-affinity aegerolysin receptor, CPE, confirmed the ability of E69A (0.06–0.5 μM) to bind CPE in the absence of cholesterol (Fig. 2A,B). The binding of E69A (0.5 μM) was enhanced ten-fold (from 200 to 2000 response units) with increasing the CPE membrane content from 1 to 5 mol%. Further, the membrane interaction of E69A was considerably stronger in the presence of PlyB, suggesting the formation of bi-component protein complexes on target CPE-containing membranes. The wild type protein, OlyA6, could not bind to any of these CPE-containing membranes lacking cholesterol, neither alone nor combined with PlyB (Fig. 2A,B). When the experiment was repeated using LUVs composed of equimolar proportions of POPC and cholesterol, supplemented with 1 or 5 mol% CPE, binding of both aegerolysins (0.03–0.5 μM) was observed (Fig. 2C,D), with E69A showing slightly higher avidity to target membranes than OlyA6. In this case also, the binding of E69A to these LUVs was several folds higher than the binding to cholesterol-free vesicles. The binding of both aegerolysins was strongly enhanced in the presence of their protein partner, PlyB.

#### Permeabilization of CPE-containing artificial membranes

**Figure 3:**
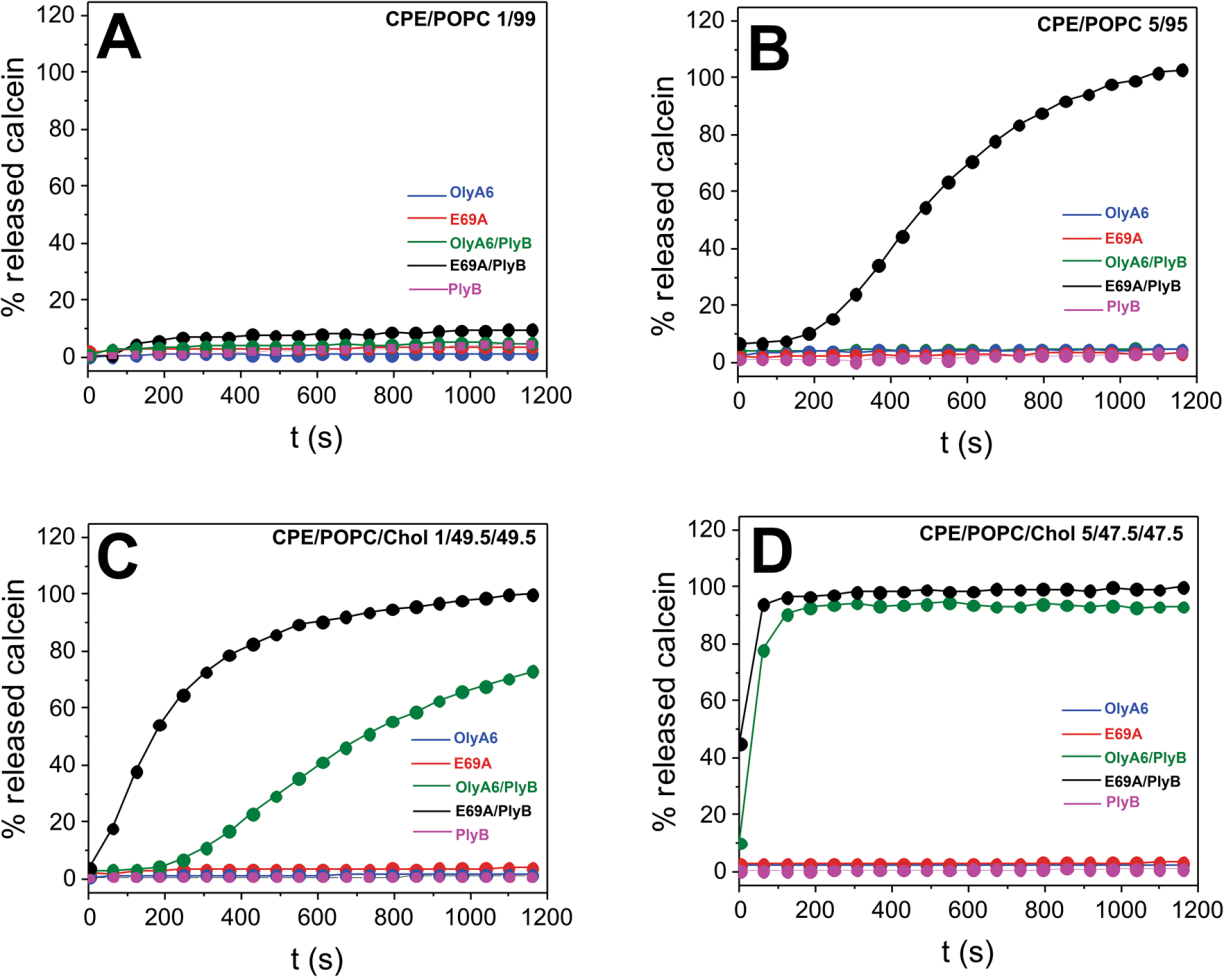
Permeabilization of small unilamellar vesicles composed of various molar proportions of CPE, POPC and cholesterol by OlyA6/PlyB and E69A/PlyB. Fluorescence intensity of calcein released from the lipid vesicles, monitored as described in the “Methods”. (**A**,**B**) Permeabilization of cholesterol-free lipid vesicles containing 1 (**A**), or 5 mol% CPE (**B**) by 60 nM aegerolysins, 40 nM PlyB, or by aegerolysins/PlyB in a 12.5/1 molar ratio. (**C**,**D**) Permeabilization of cholesterol-containing lipid vesicles supplemented with 1 (**C**), or 5 mol% CPE (**D**) by 60 nM aegerolysins, 40 nM PlyB, or by aegerolysins/PlyB in a 12.5/1 molar ratio. Mean values of triplicate analyses, where the standard error did not exceed 5%, are shown. Lipid molar proportions are indicated on the graphs. *CPE* ceramide phosphoethanolamine, *POPC* 1-palmitoyl-2-oleoyl-*sn*-glycero-3-phosphocholine, *Chol* cholesterol.

The membrane-permeabilizing activity of aegerolysins, alone or combined with PlyB, was assayed by monitoring the fluorescence of calcein released from small unilamellar vesicles having the same CPE content as in the single-cycle kinetics membrane binding assay. Aegerolysins alone (30–500 nM) or PlyB (40 nM) alone did not have any membrane-disrupting activities, and vesicle permeabilization could be observed only upon the addition of both proteins, aegerolysin and its MACPF partner (Fig. 3, Fig. S4). Permeabilization of cholesterol-free vesicles was observed exclusively with the E69A/PlyB (12.5/1, mol/mol) complex, and not with the OlyA6/PlyB (12.5/1, mol/mol) complex. This membrane-disrupting potential of E69A/PlyB depended on the membrane CPE content, as the protein complex could not permeabilize vesicles supplemented with 1 mol% CPE neither at the highest tested concentration (500 nM) (Fig. S4A), but caused 100% lysis of vesicles containing 5 mol% CPE at low nanomolar concentrations (Fig. S4B). The addition of cholesterol to calcein-loaded vesicles significantly enhanced the membrane-disruption potential of E69A/PlyB, and also enabled membrane permeabilization activity by the OlyA6/PlyB complex (Fig. 3C,D, Fig. S4C,D). This membrane permeabilization by OlyA6/PlyB and E69A/PlyB complexes was observed even with low (1 mol%) membrane concentration of CPE.

### E69A interaction with sphingolipid-containing biological membranes

**Figure 4:**
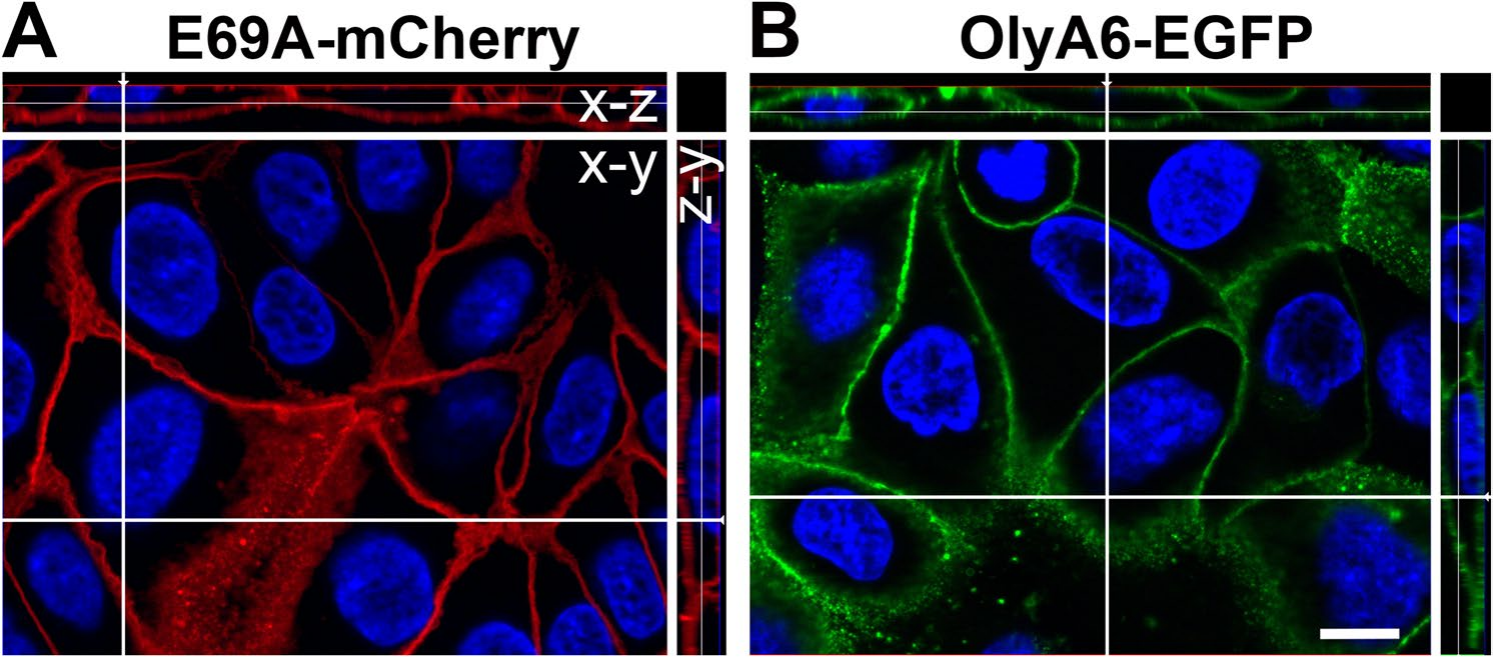
Labelling of MDCK cell membranes by E69A-mCherry and OlyA6-EGFP. MDCK cells were fixed and incubated 1 min with 1 µM E69A-mCherry (**A**) or 5 min with 2 µM OlyA6-EGPF (**B**). Nuclei are stained with DAPI (blue). Scale bar: 10 µm.

During the optimization of the protocol for labelling MDCK cells with fluorescent OlyA6 and E69A variants (E69A-mCherry or OlyA6-EGFP), it became clear that the fluorescently tagged mutant protein, E69A-mCherry, had considerably higher avidity for the target cell membranes. Incubation of MDCK cells with 1 µM E69A-mCherry for 1 min labelled the plasma membrane of all cells (Fig. S5A). Increasing the time of incubation to 5 min resulted in very intense labelling (Fig. S5C), while incubation with 2 µM E69A-mCherry for 5 min gave oversaturated labelling of membranes (Fig. S5E). When MDCK cells were incubated under the same conditions with the fluorescently tagged wild-type protein, OlyA6-EGPF, effective labelling could not be achieved with 1 µM protein concentration, applied either for 1 or 5 min (Fig. S5B,D). Only after incubation with 2 µM OlyA6-EGPF for 5 min (Fig. S5F) some cells were labelled to similar extent compared to the labelling with 1 µM E69A-mCherry for 1 min. To this end, we used these combinations of concentrations and the incubation times (1 µM E69A-mCherry for 1 min and 2 µM OlyA6-EGPF for 5 min) in our further experiments. Under these conditions, the intensity and location of the labelling by E69A-mCherry (Fig. 4A) and OlyA6-EGFP (Fig. 4B) in the plasma membrane of MDCK cells was comparable.

**Figure 5:**
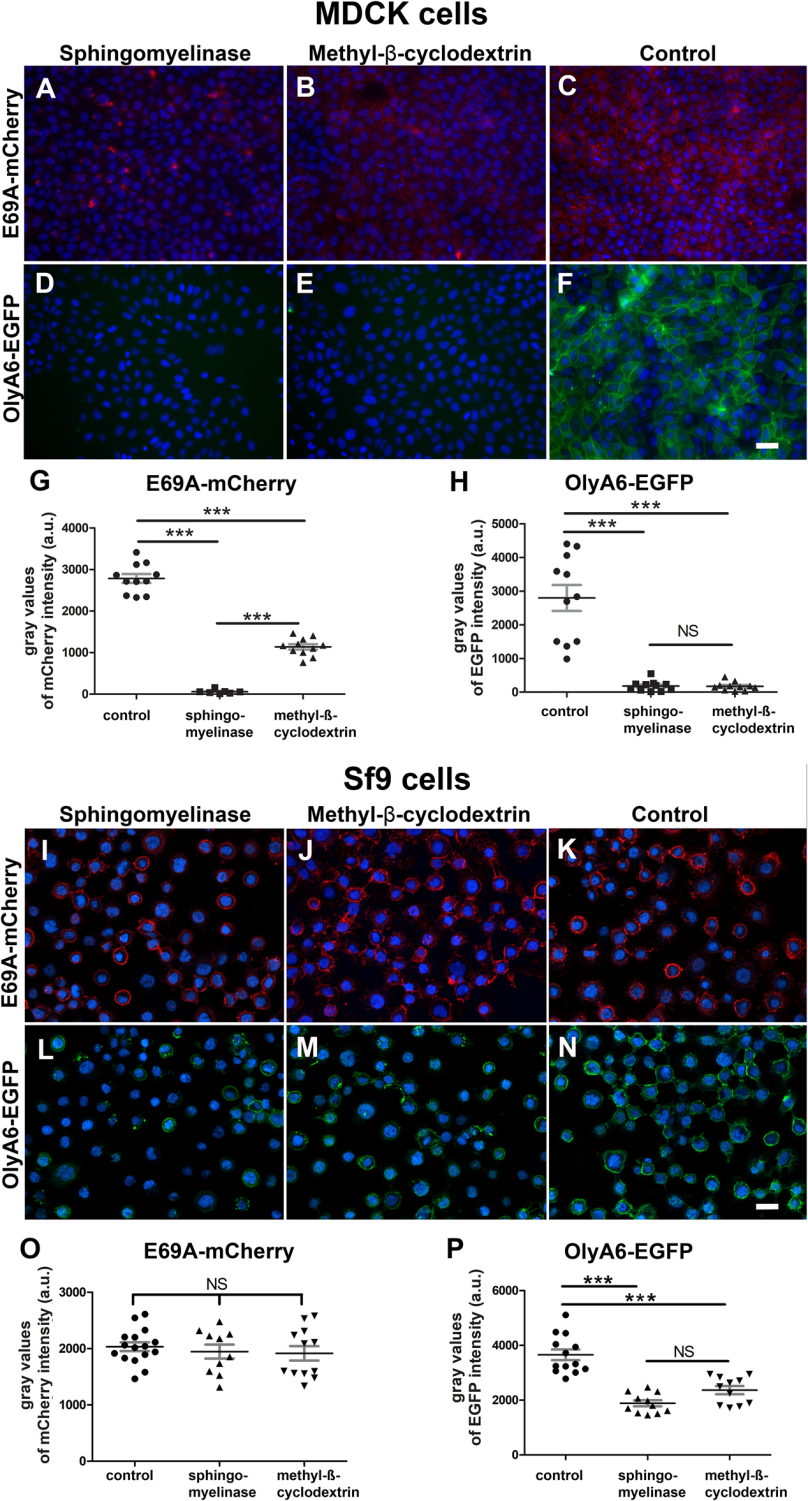
Effects of methyl-β-cyclodextrin and sphingomyelinase on binding of E69A-mCherry or OlyA6-EGFP to mammalian MDCK and insect Sf9 cells. Representative fluorescence images of MDCK cells showing reduced binding of 1 µM E69A-mCherry after a 30-min incubation of cells with 2 U/mL sphingomyelinase (**A**) or after incubation of cells with 5 mM methyl-β-cyclodextrin for 1 h (**B**), and no binding of 2 µM OlyA6-EGFP under the same conditions (**D**,**E**). Control cells are uniformly labelled by both proteins (**C**,**F**). Representative fluorescence images of Sf9 cells showing comparable binding of 1 µM E69A-mCherry after a 30-min incubation of cells with 0.5 U/mL sphingomyelinase (**I**) or after incubation of cells with 5 mM methyl-β-cyclodextrin (**J**) with control cells (**K**). The binding of 2 µM OlyA6-EGFP after sphingomyelinase (**L**) or methyl-β-cyclodextrin (**M**) treatment was reduced in comparison to control cells (**N**). Quantification of grey values of fluorescence intensities of E69A-mCherry in MDCK cells (**G**) and in Sf9 cells (**O**) and OlyA6-EGFP in MDCK cells (**H**) and in Sf9 cells (**P**) after sphingomyelinase and methyl-β-cyclodextrin treatment. Presented are averages ± SEM, ***P < 0.001, *NS* not significant. Nuclei are stained with DAPI (blue). Scale bars: (**A**–**F**) 50 µm; (**I**–**N**) 20 µm.

Pre-treatment of MDCK cells with both sphingomyelinase and methyl-β-cyclodextrin significantly reduced E69A-mCherry binding (Fig. 5A,B) and almost completely abolished the binding of OlyA6-EGFP (Fig. 5D,E) in comparison to untreated cells (Fig. 5C,F). The quantification of fluorescence intensity revealed a 98% and 59% reduction of E69A-mCherry binding after sphingomyelinase or methyl-β-cyclodextrin treatment, respectively (Fig. 5G). OlyA6-EGFP binding was reduced by 94% with both treatments under the same conditions (Fig. 5H). In Sf9 cells, E69A-mCherry binding was detected in sphingomyelinase and methyl-β-cyclodextrin treated cells (Fig. 5I,J) as intense as in untreated cells (Fig. 5K). Indeed, the quantification of fluorescence intensity revealed only 5% and 6% reduction of E69A-mCherry binding after cell treatment with sphingomyelinase and methyl-β-cyclodextrin, respectively, in comparison to untreated cells (Fig. 5O). In contrast, OlyA6-EGFP binding was decreased after sphingomyelin and cholesterol depletion from Sf9 cells (Fig. 5L,M) in comparison to untreated cells (Fig. 5N). OlyA6-EGFP binding was reduced by 48.0% after sphingomyelinase treatment and by 35.0% after methyl-β-cyclodextrin treatment, respectively (Fig. 5P).

**Figure 6:**
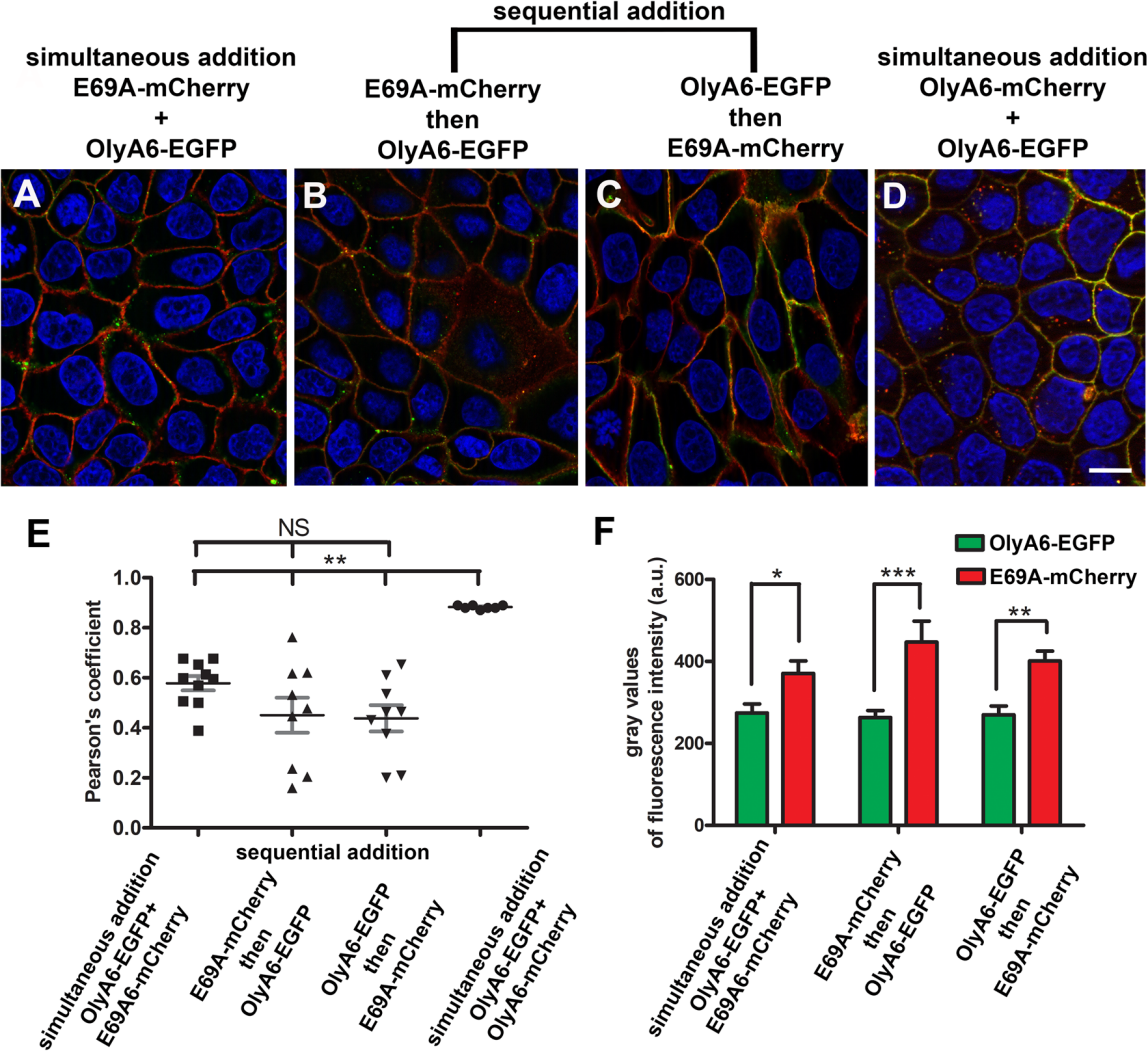
Colocalization of fluorescently tagged OlyA6 and E69A in the plasma membrane of MDCK cells. E69A-mCherry and OlyA6-EGFP were incubated with cells simultaneously (**A**), or sequentially by applying (**B**) first E69A-mCherry and then OlyA6-EGFP or (**C**) first OlyA6-EGFP and then E69A-mCherry. Two fluorescent variants of the wild-type protein (OlyA6-mCherry and OlyA6-EGFP) were incubated simultaneously (**D**). Images of colocalizations were acquired at the same experimental settings (filter sets, camera setting, and exposure time). Nuclei are stained with DAPI (blue). Scale bar: 10 µm. (**E**) The quantification of colocalization after simultaneous and sequential incubation of proteins was measured on optical sections and presented as Pearson’s coefficient. (**F**) The quantification of OlyA6-EGFP and E69A-mCherry fluorescence (grey values of fluorescence intensity) after simultaneous and sequential incubation of proteins, measured on optical sections. Presented are mean ± SEM, *P < 0.05, **P < 0.01, ***P < 0.001.

For colocalization studies, E69A-mCherry and OlyA6-EGFP were incubated with MDCK cells simultaneously or were added sequentially to determine whether E69A can occupy the binding site of OlyA6 and vice versa, or if proteins compete for the same binding site. When applied simultaneously (Fig. 6A), the degree of colocalization calculated from the Pearson’s value correlation was 0.58 ± 0.03 (Fig. 6E). Sequential incubation by exposing cells first to E69A-mCherry and then to OlyA6-EGFP (Fig. 6B) resulted in 0.45 ± 0.03 colocalization degree (Fig. 6E), and the colocalization degree value was 0.44 ± 0.05 when the sequence of the protein application was opposite (Fig. 6C). These colocalization degrees were not significantly different irrespective of application setup, however they were in a considerable contrast with the colocalization degree of the two fluorescent variants of the wild-type protein (OlyA6-mCherry and OlyA6-EGFP) (Fig. 6D). Here, the colocalization degree was strong and resulted in a value of 0.88 ± 0.003 (Fig. 6E). The quantification of EGFP and mCherry fluorescence intensity in our colocalization studies revealed that E69A-mCherry binding significantly exceeds the binding of OlyA6-EGFP irrespective of the application modality (Fig. 6F). These results confirm a high affinity of E69A to biological membranes.

### E69A/PlyB toxicity

#### Toxicity of OlyA6/PlyB and E69A/PlyB complexes to mammalian and insect cell lines

**Figure 7:**
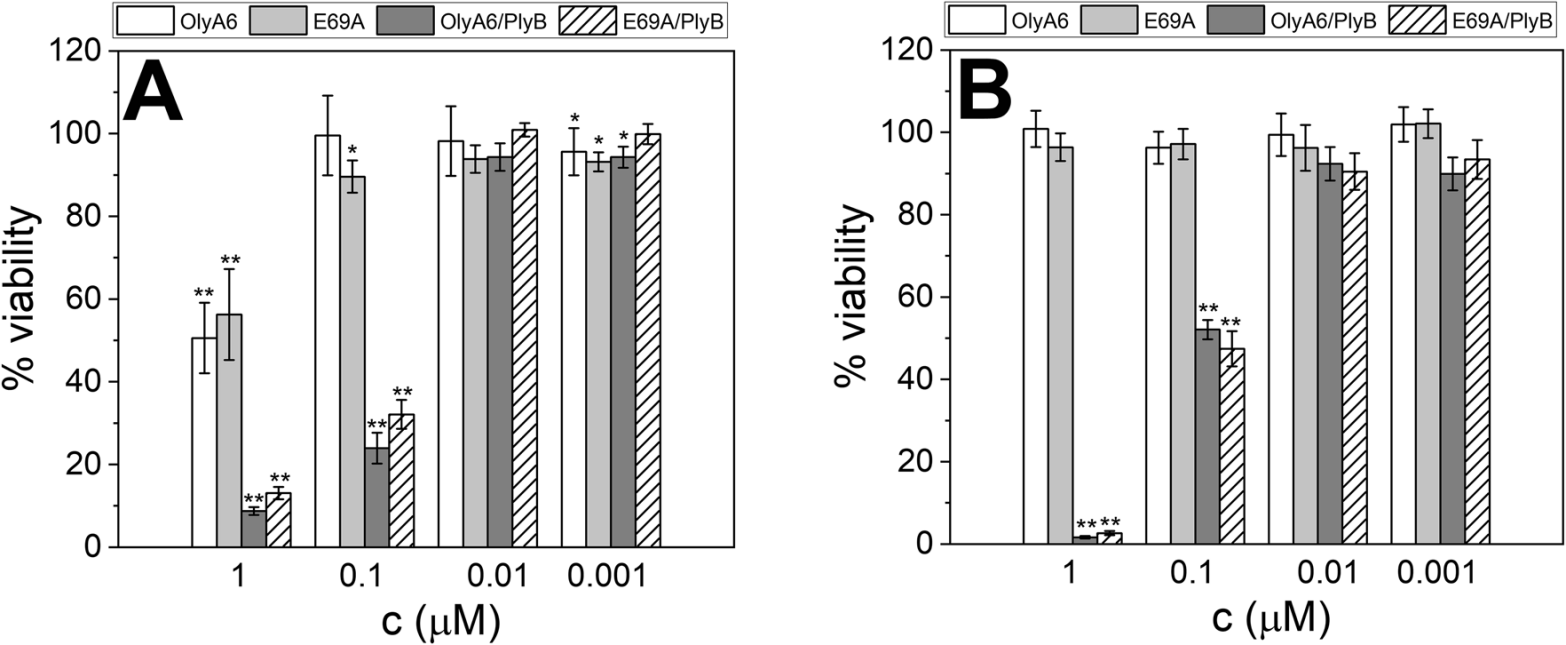
Effects of OlyA6 and E69A, alone or in combination with PlyB, on survival of insect Sf9 (**A**) and mammalian MDCK (**B**) cells. Survival rates of cells treated with OlyA6, E69A, OlyA6/PlyB and E69A/PlyB (as indicated) are expressed as the ratio between the luminescence of the treated and the control cells (in %). Data are mean ± SEM of three independent experiments (*P < 0.05; **P < 0.001).

In line with the permeabilization experiments using CPE-enriched artificial lipid vesicles, aegerolysin/PlyB complexes (in 1 μM and 0.1 μM final concentrations) were toxic to the Sf9 cells that were previously shown to contain ~ 4 mol% CPE species^12^. Both protein complexes showed similar toxicities, with 24% and 32% cells remaining viable after a 30-min incubation with 0.1 μM OlyA6/PlyB or E69A/PlyB, respectively (Fig. 7A). OlyA6 and E69A also showed an effect on Sf9 cells survival when applied alone at the highest tested concentration (1 μM), and the ratio of viable cells in these experiments was 51% and 57%, respectively. MDCK cells were not permeabilized by OlyA6 or E69A alone, but were susceptible to aegerolysin/PlyB mixtures, with 52% and 47% cells remaining viable after a 30-min incubation with 0.1 μM OlyA6/PlyB or E69A/PlyB, respectively (Fig. 7B).

#### Toxicity assay on Colorado potato beetle larvae

**Figure 8:**
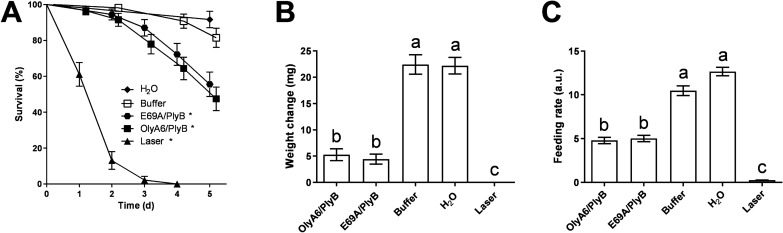
Kaplan–Meier survival curves (**A**), weight change (**B**) and feeding rate (**C**) of Colorado potato beetle larvae following exposure to aegerolysin/PlyB complexes, negative (buffer and H_2_0) and positive control (Laser) over the 5-day bioassay. Asterisk (*) denotes significant difference from control treatment (i.e., buffer) in ‘(**A**)’, whereas different lowercase letters above bars denote significant differences between treatments in ‘(**B**)’ and ‘(**C**)’ (*P < 0.05). Data points are nudged ± 0.2 units to prevent overlapping in ‘(**A**)’. H_2_O—leaf discs treated with plain tap water; Laser—positive control, insecticide Laser Plus, based on active ingredient spinosad.

Exposure of CPB larvae to leaf discs that were treated with the OlyA6/PlyB or E96A/PlyB complexes (9.0 µg/cm^2^) or with the insecticide (0.1% Laser Plus) had significant effects on their survival (χ^2^ = 255; P < 0.0001) over the 5-day bioassays. Both OlyA6/PlyB and E96A/PlyB complexes as well as the insecticide significantly increased larval mortality. Buffer-treated group did not significantly differ from water-treated leaf disc group mortality. Both protein complexes at tested concentration 9.0 µg/cm^2^ caused similar mortalities, with 47% and 55% CPB larvae surviving the 5-d bioassay after feeding on OlyA6/PlyB- or E69A/PlyB-treated leaf discs, respectively. The hazard ratio was slightly higher in the OlyA6/PlyB (3.50), compared to E69A/PlyB-treated group (2.76). Laser group had 0% survival at day 5, and a hazard ratio of 10 (Fig. 8A). The aegerolysin/PlyB complexes also exhibited significant sublethal effects on the CPB larval feeding, as indicated by the significantly smaller larval weight increase (F_4,134_ = 6.92, P < 0.0001; Fig. 8B) and feeding rate (K–W statistic_5,264_ = 197, P < 0.0001). The two protein complexes (OlyA6/PlyB vs*.* E69A/PlyB) did not differ significantly in their effect on the CPB feeding rate, resulting in a statistically undistinguishable weight change differences of the surviving larvae in the 5-d bioassays (Fig. 8C).

## Discussion

Proteins from the aegerolysin family produced by the mushroom genus *Pleurotus* were recently demonstrated to exhibit interesting biomedical and biotechnological potential^14^. It was first reported that they specifically interact with lipid rafts, membrane nanodomains of mammalian cells highly enriched in sphingomyelin and cholesterol, which led towards their exploitation as molecular tools for studying these important membrane domains^2,15^. These findings have also highlighted their potential use in treatment of some diseases in which cells contain increased numbers of raft-like domains in their membranes, as in colorectal cancer^16^, and in combating obesity and related metabolic disorders^17,18^. Targeting of lipid rafts by a native isolate from *P. ostreatus*, containing a mixture of OlyA6 and PlyB proteins has also been shown to be an excellent approach for selective elimination of urothelial cancer cells in vitro^19^.

The selectivity of OlyA6 for lipid rafts originates from its specific ability to sense the cholesterol-sequestered conformation of sphingomyelin, and this selectivity can be abolished by a single point mutation, E69A, rendering the protein able to sense sphingomyelin both in its free, and in its cholesterol-bound conformation^9^. Comparing to OlyA6, this mutant differs in side chain orientation of three amino acids within 5 Å of the center of the shallow channel responsible for the interaction with the ceramide backbone^9^. These differences probably dictate the much higher interaction of the mutant with membranes containing sphingolipids, namely sphingomyelin and CPE, as also observed in our surface plasmon resonance experiments using sphingolipid-containing membrane vesicles.

Application of the fluorescently tagged wild-type protein and its mutant E69A to the membranes of sphingomyelin-containing mammalian MDCK cells confirmed these results obtained with artificial membrane systems, and clearly showed different binding intensities of the two proteins. The colocalization degree between the wild-type and the mutant protein was nearly two-folds lower than the one obtained after incubating the cells with two different fluorescent variants of the wild-type OlyA6, suggesting that OlyA6 and E69A share only a part of their binding sites. The binding of the mutant was considerably higher than the binding of the wild type in all experimental setups, further indicating that the mutant can recognize and bind additional binding sites on the membrane. Finally, the depletion of the cell membrane sphingomyelin aborted the binding of both proteins, while the membrane cholesterol reduction almost completely (94%) abolished the binding of OlyA6-EGFP, but reduced only ~ 59% of the E69A-mCherry signal. These combined results indicate that only a part of E69A is in close association with cholesterol/sphingomyelin domains that are exclusive targets for OlyA6^2,9^. The remaining E69A-mCherry-labelled regions probably represent one or several different cholesterol-free sphingomyelin membrane pools, in which sphingomyelin can exist in monomeric form, or can form clusters with other sphingomyelin molecules and/or with glycosphingolipids^20^. These different membrane sphingomyelin pools can be distinguished by using recently discovered fluorescently labelled sphingomyelin-binding proteins^20^. For example, pore-forming proteins equinatoxin II from a sea anemone and lysenin from an earthworm label sphingomyelin monomers and homomeric sphingomyelin clusters, respectively^21,22^. However, equinatoxin II can also bind sphingomyelin associated with glycosphingolipids^23^, and lysenin can interact with sphingomyelin/cholesterol clusters^24^. On the other hand, *Pleurotus* aegerolysins, and another mushroom-derived protein, nakanori, selectively label sphingomyelin/cholesterol domains, and do not associate with other sphingomyelin membrane forms^2,15,25^. The exact nature of cholesterol-free membrane sphingomyelin pool(s) that can be sensed by E69A remains to be further explored.

Besides binding to sphingomyelin/cholesterol membrane domains, *Pleurotus* aegerolysins can bind even more specifically to artificial lipid vesicles composed of mixtures of cholesterol and the main invertebrate membrane lipid, CPE^4,26^, while they cannot bind to the cholesterol-free membranes supplemented with this sphingolipid^4^. This discovery has led to the use of *Pleurotus* aegerolysins-based cytolytic complexes as novel biopesticides for the control of herbivorous coleopteran pests^4^. Due to their ability to act through binding to a specific membrane lipid receptor, and not membrane protein(s) that are prone to mutations, the chances that the insects will evolve resistance to aegerolysin-based insecticidal protein complexes were speculated to be significantly lower. These protein complexes are also readily digested by mammalian digestive enzymes^27^, and are safe for humans and for the environment^28^. Aegerolysin proteins, and their mutants with enhanced affinity for lipid membranes, therefore represent an appealing source for developing new biopesticides.

Indeed, our experiments using CPE-enriched model lipid membranes, presented in this paper, clearly show that the E69A mutation strongly increases the binding of OlyA6 to CPE or SM-containing membranes, and also abolishes the requirement for membrane cholesterol for successful protein binding. These results corroborate the previous findings of Endapally et al.^9^ showing the avidity of E69A for different membrane conformations of sphingomyelin. This enhanced binding of E69A to membranes with low or no cholesterol content is very important, as most insects (especially at the immature stages of their development) are cholesterol auxotrophs, and their sterol membrane content is usually very low^11,29^. Furthermore, this may indicate the possible presence of different conformations of CPE in lipid membranes that OlyA6 can distinguish, as in the case of sphingomyelin. The depletion of the Sf9 cell membrane sphingomyelin or cholesterol did not significantly affect the binding of E69A-mCherry. Sf9 cells have about 4 mol% CPE and 1.5 mol% sphingomyelin in their membranes^12^. Since CPE is not a sphingomyelinase substrate^30^, the membranes of these cells, even with treated with sphingomyelinase, still harbour a considerable amount of CPE molecules that serve as high-affinity E69A receptors. This can also explain the decreased, but not completely abolished binding of OlyA6-EGFP, that requires cholesterol-bound sphingomyelin or CPE for effective membrane binding. The cholesterol membrane content of Sf9 cells is very low (0.4%, w/w^12^), so their treatment with methyl-β-cyclodextrin did not significantly abolish the binding of E69A, which can bind cholesterol-free CPE or sphingomyelin anyway. Interestingly, the binding of the wild type protein, OlyA6-EGFP, was reduced by 35% after a cholesterol depletion, indicating that in these cells some other lipid molecule could act similarly to cholesterol and induce a change in sphingolipid conformation that can be recognized by OlyA6.

In spite of these binding results obtained using the artificial and biological membranes, the cytotoxicity of E69A/PlyB complexes towards Sf9 cell line was not significantly different as compared to OlyA6/PlyB. The same trend could also be seen in toxicity tests where CPB larvae were exposed to OlyA6/PlyB and E69A/PlyB. Both aegerolysin-based cytolytic complexes caused a significant increase of mortality and decrease of larval weight gain over the 5-day bioassays, but there were no significant differences between the action of these two complexes. The LT_50_ of these protein complexes was 5 days, and the LC_50_ approximately 9 µg of proteins per 1 cm^2^ of potato leaf, which corroborates our previous results^4^. In comparison with other proteinaceous insecticidal Cry toxins of bacterial (*Bacillus thuringiensis*) origin, this activity of OlyA6/PlyB and E69A/PlyB complexes against CPB larvae is somehow lower than the activity of the Cry7Aa2 from *Bacillus thuringiensis* evaluated 4 days post-treatment^31^, but considerably higher than the activity of commercially used Cry34Ab1/Cry35Ab1 toxins who exhibited a LC_50_ at concentrations^32^ above 35 μg/cm^2^. The mortality of CPB larvae caused by OlyA6/PlyB and E69A/PlyB complexes could probably be attributed to pore-forming activity of aegerolysin-based complexes on insect intestinal cells, and the consequent colloidal-osmotic cellular lysis. Indeed, when the western corn rootworm larvae were fed OlyA6/PlyB, the midgut wall columnar epithelium showed vacuolization of the cell cytoplasm, swelling of the apical cell surface into the gut lumen, and delamination of the basal lamina underlying the epithelium^8^. It might be that CPB larvae experience similar midgut epithelium damage, but this would need to be microscopically confirmed.

## Conclusions

Our combined results confirm that the single point mutation of glutamic acid at position 69 to alanine indeed greatly improves the interaction of OlyA6 with both mammalian- and invertebrate-principal membrane sphingolipid species, the sphingomyelin and the CPE. This mutation is however not a guarantee that more pore complexes will be formed after the addition of PlyB, as we have not seen an increase of toxicity to insect and mammalian cell lines or CPB larvae. These comparable membrane-lytic potentials of OlyA6/PlyB and E69A/PlyB suggest that the mutation does not affect the side chain orientation of amino acids responsible for the interaction of the aegerolysin protein with PlyB. According to the crystal structure of PlyA/PlyB, where PlyA is an aegerolysin sharing 94% amino acid identity with OlyA6, this interaction site for PlyB is different from the interaction site responsible for binding to membrane sphingolipids^7,9^. However, the considerable increase in binding to membrane sphingolipids, and the ability to bind to cholesterol-free membrane sphingolipids, propose the OlyA6 mutant E69A, and its fluorescent fusion analogues, as both a lipid raft marker, and a marker of free and/or clustered membrane sphingomyelin and CPE.

## Materials and methods

### Materials

#### Chemicals

All chemicals used in the present study were from Sigma–Aldrich (St. Louis, MO, USA) unless specified otherwise. Porcine brain sphingomyelin, wool grease cholesterol, 1-palmitoyl-2-oleoyl-*sn*-glycero-3-phosphocholine (POPC) and CPE were from Avanti Polar Lipids (Alabaster, AL, USA). These lipids were stored at − 20 °C and dissolved in chloroform prior to use. CPE was dissolved in 1 mL chloroform/methanol (9/1, v/v).

#### Cells

Insect cells derived from the ovarian epithelial cells of the fall army worm (*Spodoptera frugiperda*; Sf9 cells; Thermo Fisher Scientific, Waltham, MA, USA) were maintained in continuous suspension culture under serum-free conditions at 28 °C in Insect XPRESS protein-free insect cell medium with L-glutamine (Lonza, Basel, Switzerland), with agitation at 150 rpm. Madin-Darby canine kidney epithelial (MDCK) cells were routinely cultured in a growth medium Advanced Dulbecco's Modified Eagle's Medium/F12 (1/1, v/v) with 5% fetal calf serum, and 1% penicillin/streptomycin. Cells were maintained in a humidified atmosphere at 37 °C and 5% CO_2_. For experiments with OlyA6 variants, the fetal calf serum in a growth medium was replaced with 5% cholesterol-free serum (HyClone, A Perbo Science Company, Utah, USA). MDCK cells were free of mycoplasma, which was tested using MycoAlert mycoplasma detection kit (Lonza, Basel, Switzerland). The culture media and supplements were purchased from Gibco, Invitrogen (Vienna, Austria).

#### Proteins

Mutation in OlyA6 construct was generated by site-directed mutagenesis using the QuickChange II XL Site-Directed Mutagenesis Kit (Agilent, Santa Clara, CA, USA). For recombinant E69A-mCherry, the obtained E69A construct was amplified using *Nde*I (F-E69A oligonucleotide) and BamHI (R-E69A oligonucleotide) sites, whereas the gene coding for mCherry was amplified using *Bam*HI (F-mCherry oligonucleotide) and *Xho*I (R-mCherry oligonucleotide) sites. Both genes were then restricted using *Bam*HI and ligated into a single nucleotide sequence. After ligation, E69A-mCherry was amplified again using *Nde*I (F-E69A oligonucleotide) and XhoI (R-mcherry oligonucleotide) and cloned into the pET21c (+) plasmid vector. The nucleotide sequences were verified by DNA sequencing.

The OlyA6, OlyA6-EGFP, OlyA6-mCherry and Δ48PlyB (henceforth PlyB) recombinant proteins were produced as described previously^1,2,4^. Protein concentrations were determined at 280 nm using a microvolume spectrophotometer (Nanodrop2000; Thermo Fisher Scientific, Waltham, MA, USA). Protein sizes and their purity were determined using sodium dodecyl sulphate polyacrylamide gel electrophoresis (SDS-PAGE; Bio-Rad, Hercules, CA, USA) on homogeneous 12% acrylamide gels. The proteins were then stained with SimplyBlue SafeStain (Thermo Fisher Scientific, Waltham, MA, USA) (Fig. S1).

#### Target insects

The Colorado potato beetle (CPB; *Leptinotarsa decemlineata* (Say)), larvae were obtained from laboratory population reared in experimental glass house at Agriculture Institute of Slovenia. A continuous mass rearing of CPB was established under artificial conditions (22 ± 5 °C, 40–60% relative humidity, 18 h:6 h day:night photoperiod). The initial population of adult CPB was derived from Laboratory of Entomology at Fraunhofer-Institut für Molekularbiologie und Angewandte, Germany, and was amended with adult beetles collected from potato fields from central Slovenia. CPB were kept in meshed rearing cages where they fed and laid eggs on potted potato plants, grown under the same conditions as described above. After the larvae have reached the L2–L3 development stage, a total of 90 larvae were collected for each laboratory bioassay purposes, while the rest of the larvae were allowed to mature and pupate to get the next generation of adult CPB beetles.

### Methods

#### Preparation of artificial lipid vesicles

Multilamellar vesicles were prepared with different molar ratios of lipids (final concentration, 5 mg/mL) in 140 mM NaCl, 20 mM Tris, 1 mM EDTA, pH 7.4, as described in^33^. The lipid molar ratios and total lipid concentrations in lipid vesicle suspensions were determined colorimetrically using free cholesterol E and phospholipids C kits (Wako Pure Chemicals, Richmond, VA, USA). These suspensions of multilamellar vesicles were subjected to five freeze–thaw cycles and then extruded through 0.1 µm polycarbonate filters (Millipore, Burlington, MA, USA) at ~ 40 to 60 °C in order to prepare large unilamellar vesicles (LUVs). For the preparation of LUVs obtained from the lipids of the Sf9 cell line, the extraction of total lipids from this cell line was performed according to the protocol of Blight and Dyer^34^. Small unilamellar vesicles loaded with calcein at the self-quenching concentration (80 mM) were prepared by sonication of multilamellar vesicles as described previously^33^.

#### Surface plasmon resonance-based binding studies

Interactions of aegerolysins OlyA6 and E69A or complexes OlyA6/PlyB and E69A/PlyB with LUVs were monitored using a surface plasmon resonance-based refractometer (Biacore T-200; GE Healthcare, Chicago, IL, USA) and an L1 sensor chip with 20 mM Tris, 140 mM NaCl, 1 mM EDTA, pH 7.4 as running buffer. After initial cleaning of the chip with regeneration solutions of sodium dodecyl sulphate and octyl-β-d-glucopyranoside with 1-min injections at a flow rate of 10 μL/min, LUVs were bound to the second flow cell of the sensor chip to achieve responses of ~ 8000 RU. The first flow cell was left empty to control for possible nonspecific binding of the proteins to the dextran matrix of the chip.

Nonspecific binding of the proteins was minimized by injection of 0.1 mg/mL bovine serum albumin for 1 min at a flow rate of 30 μL/min. First, multi-cycle kinetic experiments were performed to test the interactions of OlyA6 and E69A (0.25, 0.5, and 1 µM) with equimolar LUVs (SM/POPC, SM/Chol, CPE/POPC, CPE/POPC/Chol). When testing the interaction with LUVs obtained from the total lipid extract of the Sf9 cell line, OlyA6 and E69A were injected at concentrations of 1, 2, 3, 4, and 5 µM.

In addition, single-cycle kinetic experiments were performed in which OlyA6, E69A, OlyA6/PlyB and E69A/PlyB (aegerolysin/PlyB molar ratio 12.5/1) were injected at aegerolysin concentrations of 0.03, 0.06, 0.12, 0.25, and 0.5 µM over LUVs containing 1 or 5 mol% CPE in the presence or absence of cholesterol, with no dissociation in between and a dissociation time of 180 s at the end.

Chip regeneration was achieved with 1-min injections of 0.5% sodium dodecyl sulphate and 40 mM β-d-glucopyranoside at a flow rate of 10 μL/min. Experiments were performed at 25 °C. Data were processed using BIAevaluation software, version 3.2.1 (GE Healthcare, Chicago, IL, USA)^35^.

#### Permeabilization of the small unilamellar vesicles

Permeabilization of calcein-loaded small unilamellar vesicles was determined using a fluorescence microplate reader (Infinite NANO, Tecan, Männedorf, Switzerland) with excitation and emission set at 485 nm and 535 nm, respectively. Calcein-loaded vesicles composed of various molar proportions of lipids were exposed to aegerolysins (concentrations range, 0–0.5 µM), PlyB (40 nM), and to aegerolysin/PlyB mixtures at a 12.5/1 molar ratio. The experiments were run for 20 min at 25 °C. The permeabilization induced by lytic aegerolysin complexes was expressed as a percentage of maximal permeabilization obtained after addition of Triton-X 100 at final concentration of 1 mM.

#### Labeling of mammalian and insect cells

After growing mammalian MDCK cells on glass coverslips (3 × 10^4^ cells/cm^2^) and insect Sf9 cells on µ-slide 8-well plates (Ibidi, Gräfelfing, Germany) for 2 days, the medium was replaced with a fresh one and the cells were fixed with 2% formaldehyde for 15 min at room temperature. The fixed cells were then washed with phosphate-buffered saline (PBS) and incubated with E69A-mCherry (1 µM) for 1 min or with OlyA6-EGFP (2 µM) for 5 min at room temperature, washed again with PBS and embedded in mounting medium with nuclei marker (Fluoroshield with DAPI, Abcam, Cambridge, UK). Alternatively, before fixation and addition of fluorescently tagged proteins, cells were incubated at 37 °C with methyl-β-cyclodextrin (5 mM) for 1 h or with *Bacillus cereus* sphingomyelinase (2 U/mL for MDCK cells and 0.5 U/mL for Sf9 cells) for 30 min in order to reduce membrane cholesterol or sphingomyelin content, respectively. Cells were imaged with AxioImager Z.1 wide-field fluorescence microscope (Zeiss, Germany) and objective 20 ×/NA 0.75 and oil objective 63 ×/NA 1.40. Optical sections were made using oil objective and ApoTome device (Zeiss, Germany). Images were acquired with AxioCam camera (Zeiss, Germany), using the same exposure time for EGFP and mCherry for treated and untreated cells.

For colocalization studies, OlyA6 fluorescent variants were applied to the cells simultaneously or sequentially. For simultaneous colocalization studies, E69A-mCherry (1 µM) and OlyA6-EGFP (2 µM) were applied to fixed cells for 5 min. In sequential colocalization studies, cells were first exposed to E69A-mCherry (1 µM) for 1 min and to OlyA6-EGFP (2 µM) for 5 min, or vice versa. Colocalization of OlyA6-EGFP and OlyA6-mCherry was performed by applying both proteins (each at a concentration of 2 µM) to the cells for 5 min. In all cases, the cells were washed with PBS after labelling and embedded in mounting medium (Fluoroshield with DAPI, Abcam, Cambridge, UK). Images were taken using a fluorescence microscope (Axio Imager Z1), using oil objective (63 ×/NA 1.40) and ApoTome device (Zeiss) for optical sections generation. Images were acquired with AxioCam camera (Zeiss). Pearson's correlation coefficient was used to quantify the colocalization between OlyA6-EGFP and E69A-mCherry, and gray values of fluorescence intensities of OlyA6-EGFP and E69A-mCherry were measured in raw images of optical sections (Zen program, Zeiss, Germany). Colocalizations were presented as mean values of Pearson’s coefficient ± SEM, and the amount of OlyA6-EFGP and E69A-mCherry in colocalization studies was presented as mean value of grey intensities ± SEM.

#### Cell viability assay

Cell viability assay was performed using the CellTiter-Glo reagent (Promega, Madison, WI, USA), following the manufacturer instructions. Sf9 and MDCK cells were plated in sterile 96-well black Costar plates (Corning, Glendale, AR, USA) at 1.5 × 10^5^ cells/cm^2^ for Sf9 cells and 3 × 10^5^ cells/cm^2^ for MDCK cells in their respective growth media. Cells were left to attach and after 24 h for Sf9 or 48 h for MDCK cells, different concentrations (0.001 µM, 0.01 µM, 0.1 µM, 1 µM) of aegerolysins or aegerolysin/PlyB (12.5/1, mol/mol) complexes in growth medium were added. After 30 min of incubation at room temperature, the content in each well was replaced with fresh growth medium and CellTiter-Glo reagent was added. The luminescence was measured using microplate reader (Cytation 3, BioTek, Vinooski, VT, USA). The results were expressed as a percentage of the luminescence of treated vs*.* untreated cells as mean ± SEM of three independent experiments.

#### Insecticidal tests

The insecticidal tests were carried in accordance with the Slovenian law on animal protection (U.l.RS, 2007). The study was not subject to ethical protocols according to EU legislature (Directive 2010/63/EU).

##### Colorado potato beetle

Colorado potato beetle 2nd instar larvae were collected from greenhouse rearing and starved for 3 h prior to experiments. The mean survival time (LT_50_) was determined by feeding the larvae on potato leaf discs 14 mm in diameter soaked in OlyA6/PlyB or E96A/PlyB (0.5 mg/mL aegerolysin, 0.04 mg/mL PlyB^4^) for 5 min. By weighing the treated leaf-discs we determined the concentration of aegerolysin/PlyB on the leaf surface to be 9.0 µg/cm^2^. From this data we were able to approximate median lethal concentration (LC_50_). A single treated leaf disc and a CPB larva were placed in each well of a six-well plate. Three replicates of six-well plates were performed for each treatment, and the experiment was repeated three times independently, resulting in a total of 54 larvae for each treatment. The bioassay was performed in a growth chamber at 22 ± 1 °C and 77% relative humidity and with a photoperiod of 12 h:12 h day:night. Leaf discs treated with a buffer mixture of the two storage buffers for the proteins from the aegerolysin/PlyB complex (20 mM Tris pH 7.4/20 mM Tris, 140 mM NaCl, 2% glycerol pH 8) in the same volume ratio as the two proteins were used as negative controls. Treatment with a 0.1% dilution of the insecticide Laser Plus (active ingredient Spinosin A + Spinosin D, 480 g/L; Dow AgroSciences V.m.b.H., Austria) was used as a positive control. Unsterile tap water was used to test for possible buffer toxicity. Survival was recorded daily for 5 days. The weight of live larvae was determined on days 1 and 5. From these data, the weight change parameter was calculated. In addition, the feeding rate of CPB larvae was evaluated using the following class system: class 0—leaf disc 0–5% eaten, class 1—leaf disc 5–25% eaten, class 2—leaf disc 25–50% eaten, class 3—leaf disc 50–90% eaten, and class 4—leaf disc 90–100% eaten. Fresh treated potato leaf discs were provided when more than 90% of the leaf disc was eaten (class 4 feeding rate).

#### Statistical analysis

The time-based progressive decrease of insect survival was analyzed using Kaplan–Meier survival analysis. When comparing multiple survival curves, the significance threshold was corrected using the Bonferroni method. From the steepness of the slope of the survival curve a parameter Hazard ratio was calculated, as a measure of how rapidly the subjects died. The hazard ratio was computed for each treatment from the survival curves, compared to the buffer (negative control).

Statistical analysis was performed using GraphPad Prism (GraphPad Software; GraphPad Software, San Diego, CA, USA, http://www.graphpad.com)^36^. Data were presented as the mean ± SEM. The effects of aegerolysin-based protein complexes on CPB larval weight changes, cell viability, the effect of methyl-β-cyclodextrin and sphingomyelinase on protein binding, and Pearson’s coefficient were analyzed using one-way ANOVA for an overall difference. If significant, this was followed by Tukey's multiple comparisons post-hoc tests, while CPB feeding rate data were analyzed using Kruskal–Wallis tests followed by Dunn's multiple comparisons post-hoc tests. When comparing multiple survival curves, the significance threshold was corrected using the Bonferroni method. Differences were considered to be statistically significant when P < 0.05 unless stated otherwise.

## Supplementary Information

Supplementary Figures.

## Data Availability

Sequences of the proteins used in this study were taken from NCBI database (accession numbers: P83467.2, 6MYI, 6MYK), and are available in the public domain.

## References

[1] Ota K (2013). Membrane cholesterol and sphingomyelin, and ostreolysin A are obligatory for pore-formation by a MACPF/CDC-like pore-forming protein, pleurotolysin B. Biochimie.

[2] Skočaj M (2014). Tracking cholesterol/sphingomyelin-rich membrane domains with the ostreolysin A-mCherry Protein. PLoS One.

[3] Butala M (2017). Aegerolysins: Lipid-binding proteins with versatile functions. Semin. Cell Dev. Biol..

[4] Panevska A (2019). Pore-forming protein complexes from *Pleurotus* mushrooms kill western corn rootworm and Colorado potato beetle through targeting membrane ceramide phosphoethanolamine. Sci. Rep..

[5] Balbi T (2022). Ceramide aminoethylphosphonate as a new molecular target for pore-forming aegerolysin-based protein complexes. Front. Mol. Biosci..

[6] Kraševec N, Skočaj M (2022). Towards understanding the function of aegerolysins. Toxins.

[7] Lukoyanova N (2015). Conformational changes during pore formation by the perforin-related protein pleurotolysin. PLoS Biol..

[8] Milijaš Jotić M (2021). Dissecting out the molecular mechanism of insecticidal activity of ostreolysin A6/pleurotolysin B complexes on western corn rootworm. Toxins.

[9] Endapally S (2019). Molecular discrimination between two conformations of sphingomyelin in plasma membranes. Cell.

[10] Johnson KA, Endapally S, Vazquez DC, Infante RE, Radhakrishnan A (2019). Ostreolysin A and anthrolysin O use different mechanisms to control movement of cholesterol from the plasma membrane to the endoplasmic reticulum. J. Biol. Chem..

[11] Guan XL (2013). Biochemical membrane lipidomics during *Drosophila* development. Dev. Cell.

[12] Novak M (2020). Binding specificity of ostreolysin A6 towards Sf9 insect cell lipids. Biochim. Biophys. Acta.

[13] Panevska A, Skočaj M, Križaj I, Maček P, Sepčić K (2019). Ceramide phosphoethanolamine, an enigmatic cellular membrane sphingolipid. Biochim. Biophys. Acta Biomembr..

[14] Panevska A, Skočaj M, Modic Š, Razinger J, Sepčić K (2021). Aegerolysins from the fungal genus *Pleurotus*—Bioinsecticidal proteins with multiple potential applications. J. Invertebr. Pathol..

[15] Bhat HB (2013). Binding of a pleurotolysin ortholog from *Pleurotus eryngii* to sphingomyelin and cholesterol-rich membrane domains. J. Lipid Res..

[16] Nimri L (2017). A recombinant fungal compound induces anti-proliferative and pro-apoptotic effects on colon cancer cells. Oncotarget.

[17] Nimri L, Staikin K, Peri I, Yehuda-Shnaidman E, Schwartz B (2018). Ostreolysin induces browning of adipocytes and ameliorates hepatic steatosis. J. Gastroenterol. Hepatol..

[18] Oren T (2017). Recombinant ostreolysin induces brown fat-like phenotype in HIB-1B cells. Mol. Nutr. Food Res..

[19] Resnik N (2015). Highly selective anti-cancer activity of cholesterol-interacting agents methyl-β-cyclodextrin and ostreolysin A/pleurotolysin B protein complex on urothelial cancer cells. PLoS One.

[20] Kobayashi T (2021). Impact of intrinsic and extrinsic factors on cellular sphingomyelin imaging with specific reporter proteins. Contact.

[21] Ishitsuka R, Yamaji-Hasegawa A, Makino A, Hirabayashi Y, Kobayashi T (2004). A lipid-specific toxin reveals heterogeneity of sphingomyelin-containing membranes. Biophys. J..

[22] Yachi R (2012). Subcellular localization of sphingomyelin revealed by two toxin-based probes in mammalian cells. Genes Cells.

[23] Makino A (2015). Visualization of the heterogeneous membrane distribution of sphingomyelin associated with cytokinesis, cell polarity, and sphingolipidosis. FASEB J..

[24] Ishitsuka R, Kobayashi T (2007). Cholesterol and lipid/protein ratio control the oligomerization of a sphingomyelin-specific toxin, lysenin. Biochemistry.

[25] Makino A (2017). A novel sphingomyelin/cholesterol domain-specific probe reveals the dynamics of the membrane domains during virus release and in Niemann-Pick type C. FASEB J..

[26] Bhat HB (2015). Evaluation of aegerolysins as novel tools to detect and visualize ceramide phosphoethanolamine, a major sphingolipid in invertebrates. FASEB J..

[27] Landi N (2022). Characterization and cytotoxic activity of ribotoxin-like proteins from the edible mushroom *Pleurotus eryngii*. Food Chem..

[28] Panevska A (2021). Effects of bioinsecticidal aegerolysin-based cytolytic complexes on non-target organisms. Toxins.

[29] Carvalho M (2012). Effects of diet and development on the *Drosophila* lipidome. Mol. Syst. Biol..

[30] Sergelius C (2012). Structure–activity relationship of sphingomyelin analogs with sphingomyelinase from *Bacillus cereus*. Biochim. Biophys. Acta Biomembr..

[31] Domínguez-Arrizabalaga M, Villanueva M, Fernandez AB, Caballero PA (2019). Strain of *Bacillus thuringiensis* containing a novel *cry7Aa2* gene that is toxic to *Leptinotarsa decemlineata* (Say) (Coleoptera: Chrysomelidae). Insects.

[32] Schlotter, P. & Storer, N. Cry34/35Ab1 mode of action and efficacy. Cost action 862, Bacterial toxins for insect control, WG5 Workshop Salzau, Kiel, Germany, 27th March 2009 (2009).

[33] Sepčić K (2003). Interaction of ostreolysin, a cytolytic protein from the edible mushroom *Pleurotus ostreatus*, with lipid membranes and modulation by lysophospholipids. Eur. J. Biochem..

[34] Bligh EG, Dyer WJ (1959). A rapid method of total lipid extraction and purification. Can. J. Biochem. Physiol..

[35] Biacore T200 Software Upgrade 3.2. https://www.cytivalifesciences.com/en/us/shop/protein-analysis/spr-label-free-analysis/software/biacore-t200-software-upgrade-3-1-p-05916 (2023).

[36] GraphPad Software. http://www.graphpad.com (2023).

