# Supplementary material for: A single point mutation expands the applicability of ostreolysin A6 in biomedicine
Source: Sci Rep. 2023 Feb 7;13:2149. doi: 10.1038/s41598-023-28949-7 (PMC9905591; doi:10.1038/s41598-023-28949-7)
Supplement: Supplementary file 1 — Supplementary Figures. [file 41598_2023_28949_MOESM1_ESM.docx]

### Supplemental Information

**A single point mutation expands the applicability of ostreolysin A6 in biomedicine**

***Running title:* The applicability of OlyA6 mutant**

Anastasija Panevska^1^, Nastja Čegovnik^1^, Klavdija Fortuna^1^, Alen Vukovič^1^, Maja Grundner^1^, Špela Modic^2^, Gregor Bajc^1^, Matej Skočaj^1^, Martina Mravinec Bohte^1^, Lara Larisa Popošek^1^, Primož Žigon^2^, Jaka Razinger^2^, Peter Veranič^3^, Nataša Resnik^3,^*, Kristina Sepčić^1,^*

^1^Department of Biology, Biotechnical Faculty, University of Ljubljana, Jamnikarjeva 101, 1000 Ljubljana, Slovenia

^2^Agricultural Institute of Slovenia, Hacquetova ulica 17, 1000 Ljubljana, Slovenia

^3^Institute of Cell Biology, Medical faculty University of Ljubljana, Vrazov trg 2, 1000 Ljubljana, Slovenia

**
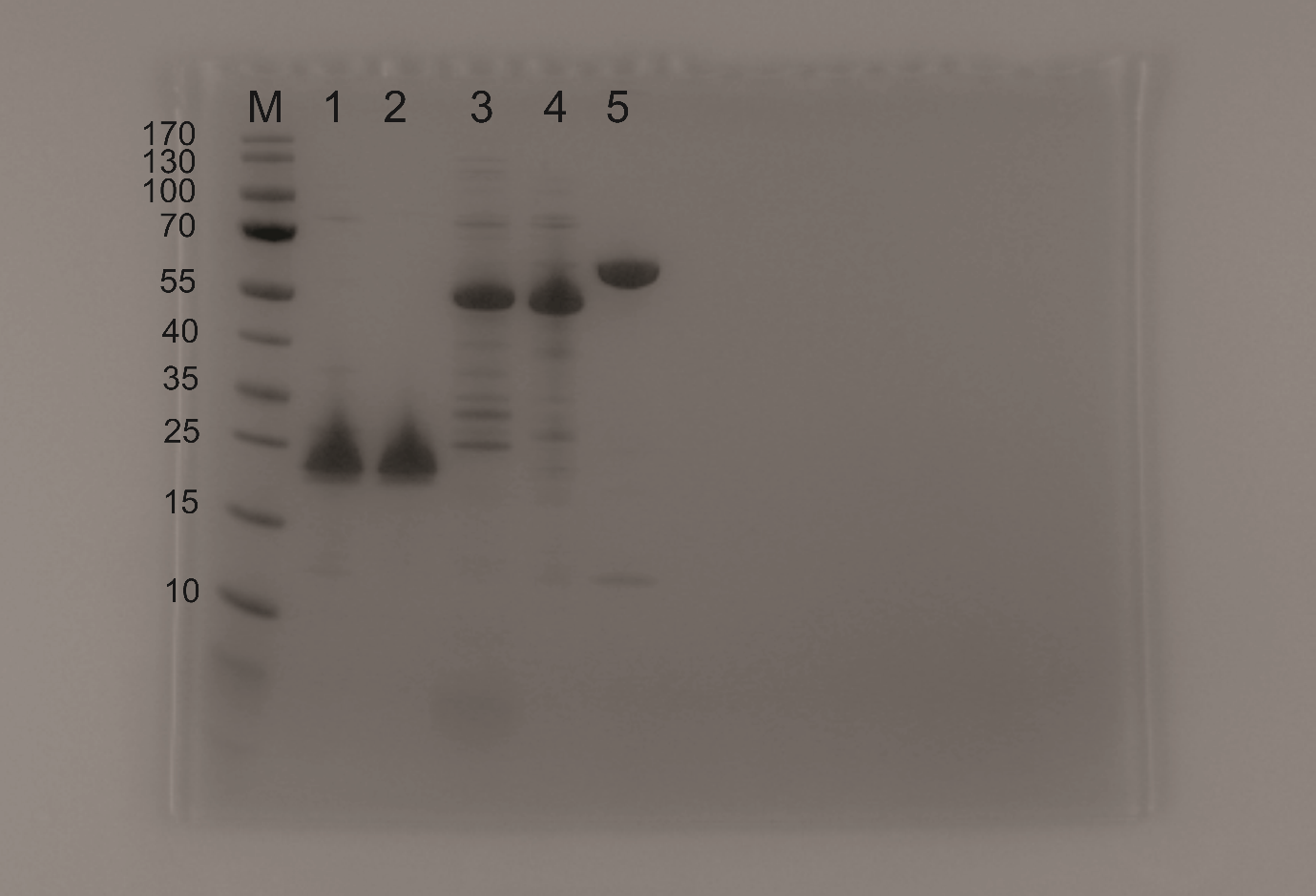
**

**Supplemental Figure S1:** **Purification of the recombinant proteins OlyA6 (lane 1), E69A (lane 2), E69A-mCherry (lane 3), OlyA6-EGFP (lane 4) and PlyB (lane 5).** Aegerolysin proteins were expressed in soluble forms, whereas PlyB was purified from inclusion bodies. All proteins were further purified with Ni-NTA affinity chromatography as indicated in the Methods. Protein fractions were subjected to SDS-PAGE and stained with SimplyBlue SafeStain (Thermo Fisher Scientific, USA). The size in kiloDaltons is shown. M = molecular weight markers.


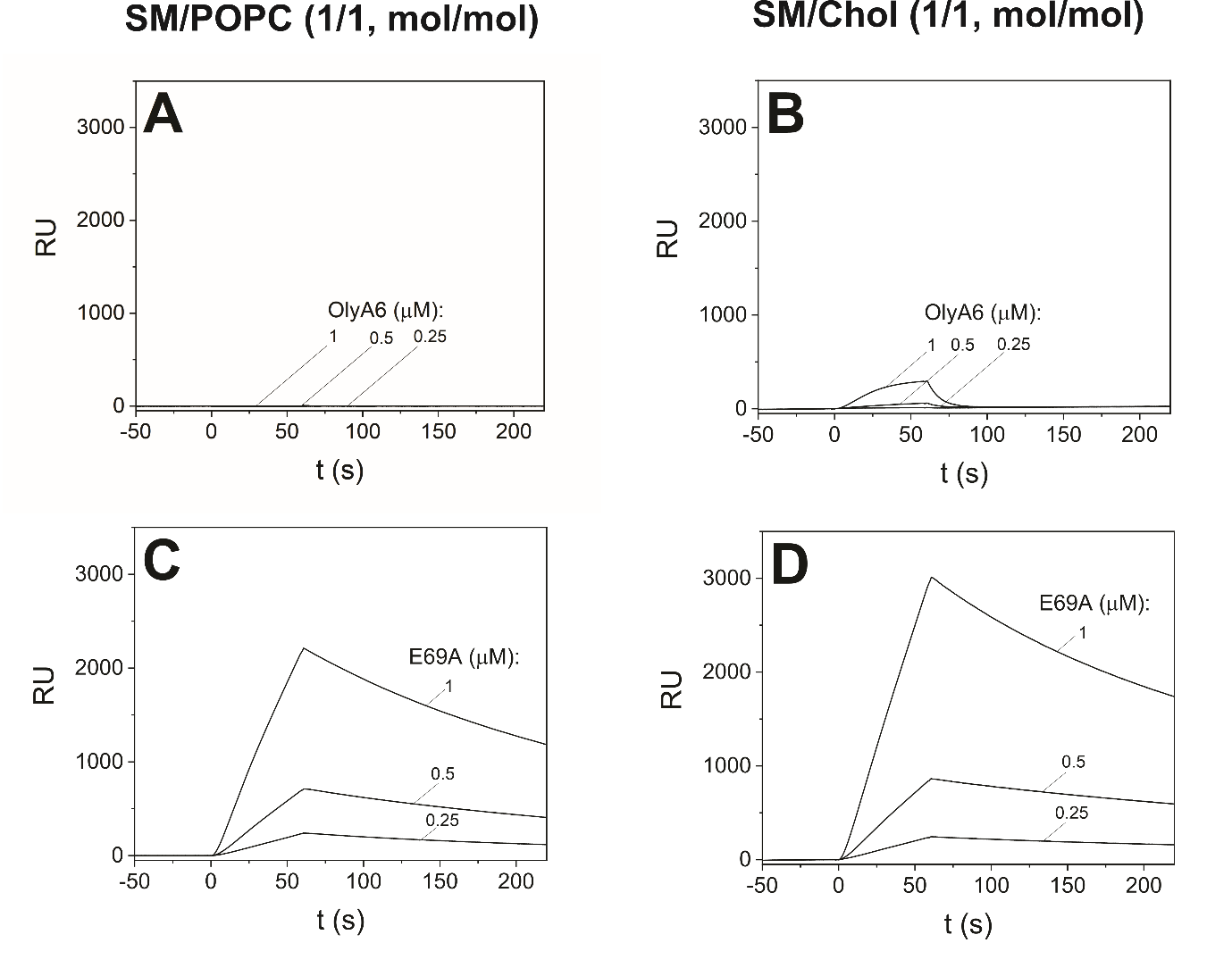


**Supplemental Figure S2. Surface plasmon resonance of the interactions of OlyA6 (A,B) and E69A (C,D), with SM/POPC (1/1, mol/mol; left panels) and SM/Chol (1/1, mol/mol; right panels) large unilamellar lipid vesicles.** The vesicles were immobilized (Biacore L1 chip) to approximately 8000 RU and the proteins were injected in running buffer (flow rate, 10 μL/min), using the kinetic titration approach in a multi-cycle by injections of 0.25, 0.5 and 1 µM concentration. Representative sensorgrams of triplicate analyses are shown. SM, sphingomyelin; POPC, 1-palmitoyl-2-oleoyl-*sn*-glycero-3-phosphocholine; Chol, cholesterol.


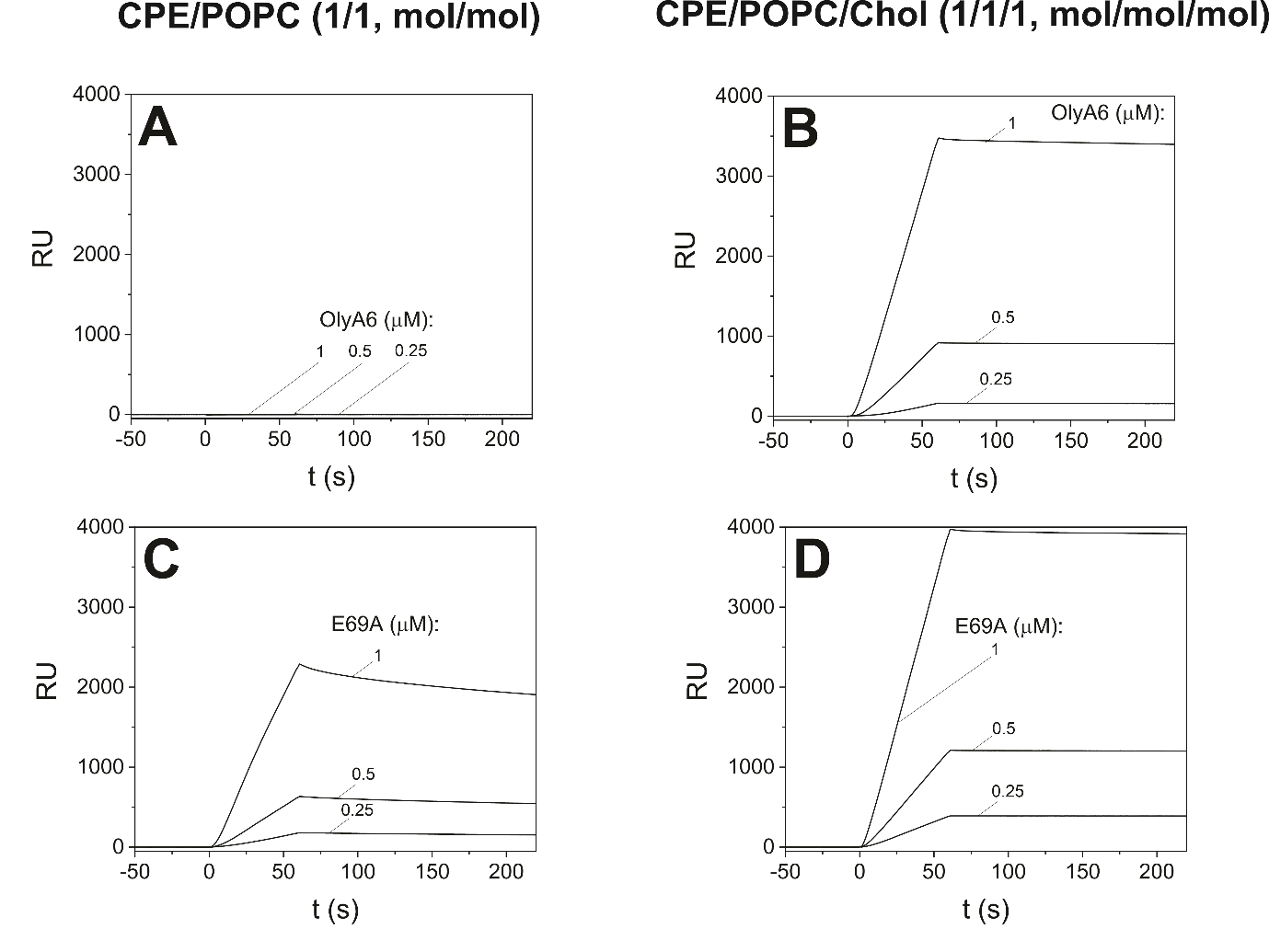


**Supplemental Figure S3. Surface plasmon resonance of the interactions of OlyA6 (A,B) and E69A (C,D), with CPE/POPC (1/1, mol/mol; left panels) and CPE/POPC/Chol (1/1/1, mol/mol/mol; right panels) large unilamellar lipid vesicles.** The vesicles were immobilized (Biacore L1 chip) to approximately 8000 RU and the proteins were injected in running buffer (flow rate, 10 μL/min), using the kinetic titration approach in a multi-cycle by injections of 0.25, 0.5 and 1 µM concentration. Representative sensorgrams of triplicate analyses are shown. CPE, ceramide phosphoethanolamine; POPC, 1-palmitoyl-2-oleoyl-*sn*-glycero-3-phosphocholine; Chol, cholesterol.


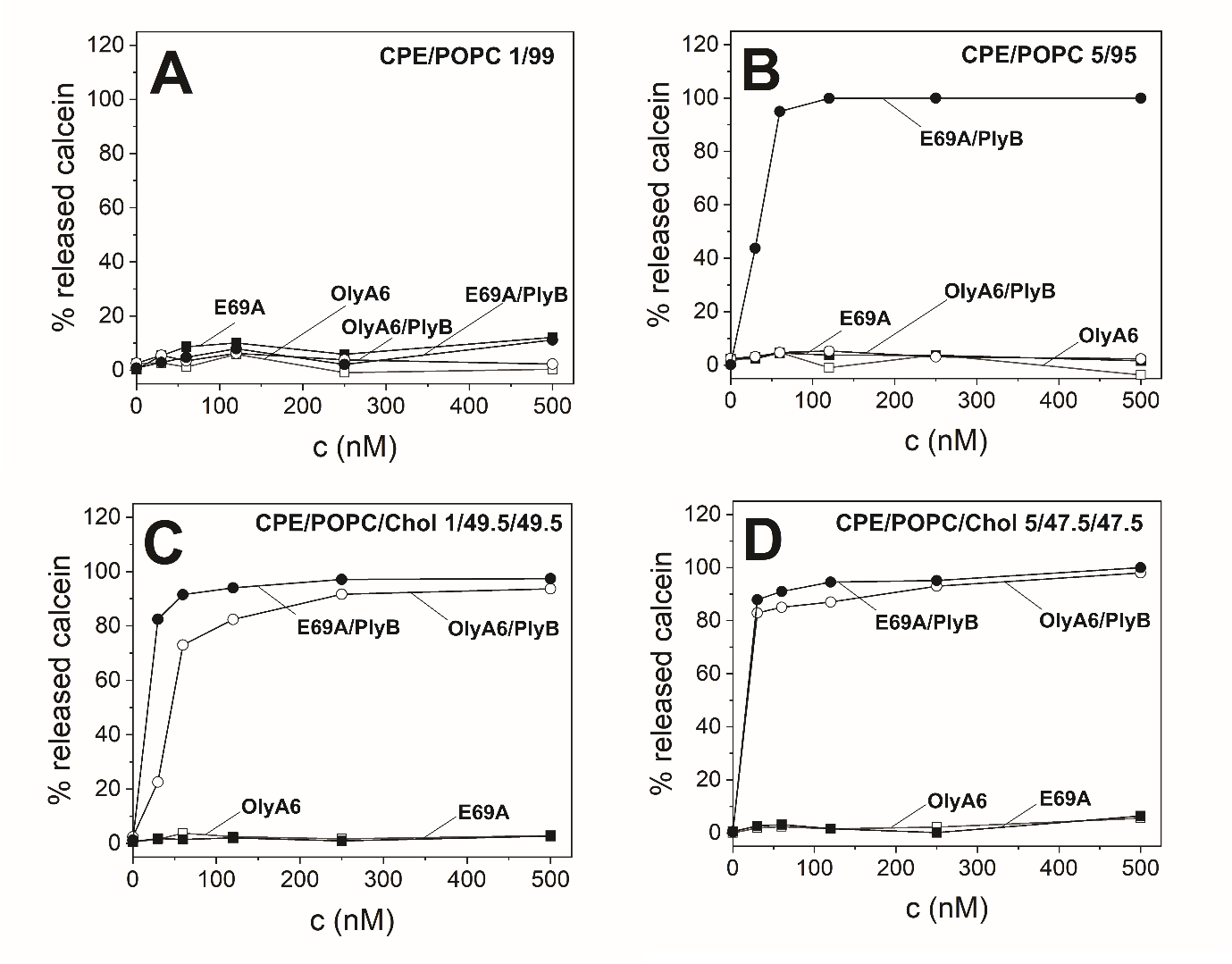


**Supplemental Figure S4. Concentration-dependent permeabilization of small unilamellar vesicles composed of various molar proportions of CPE, POPC and cholesterol by OlyA6/PlyB and E69A/PlyB**. Fluorescence intensity of calcein released from the lipid vesicles, monitored as described in the Methods. Mean values of triplicate analyses, where the standard error did not exceed 5%, are shown. (**A, B**) Permeabilization of cholesterol-free lipid vesicles containing 1 (**A**), or 5 mol% CPE (**B**) by 0-500 nM aegerolysin with PlyB. (**C, D**) Permeabilization of cholesterol-containing lipid vesicles supplemented with 1 (**C**), or 5 mol% CPE (**D**) by 0-500 nM aegerolysin with PlyB. Aegerolysin/PlyB molar ratio = 12.5/1. Lipid molar proportions are indicated on the graphs. CPE, ceramide phosphoethanolamine; POPC, 1-palmitoyl-2-oleoyl-*sn*-glycero-3-phosphocholine; Chol, cholesterol.


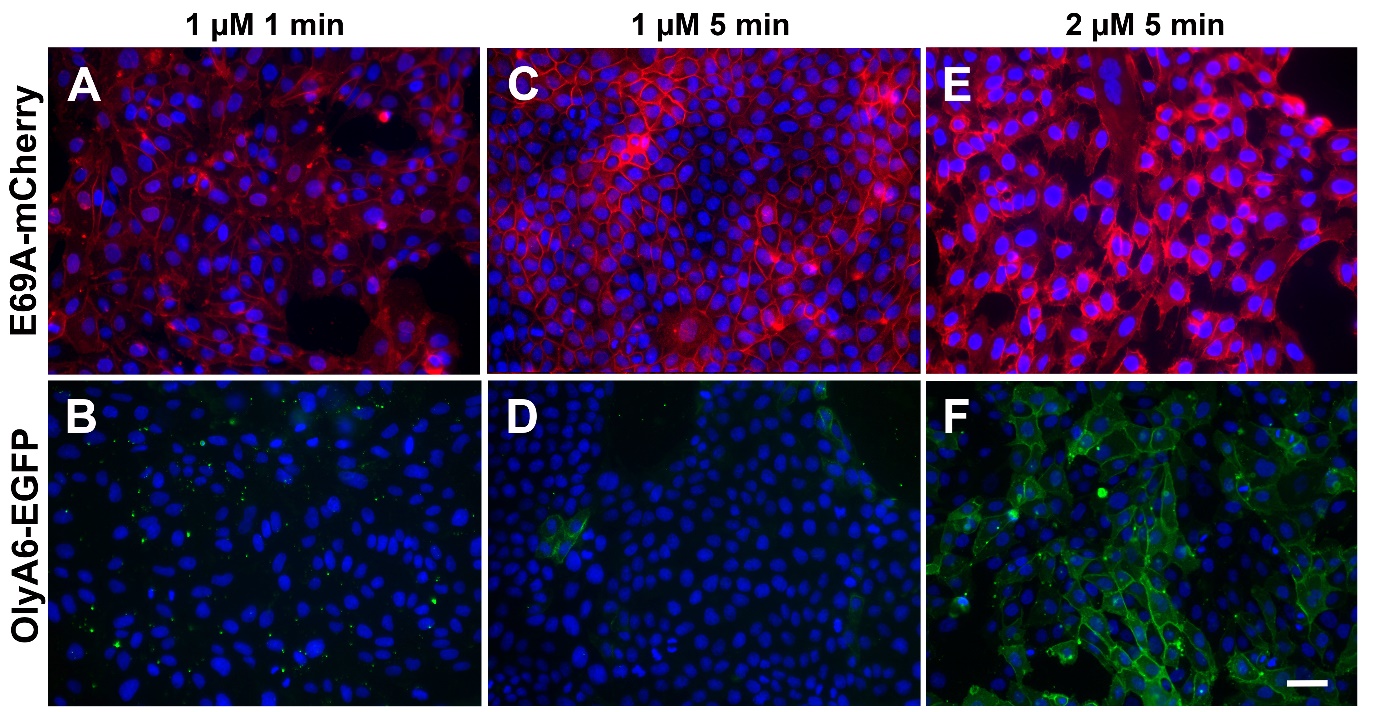


**Supplemental Figure S5. Labelling of MDCK cells with E69A-mCherry (upper line) and OlyA6-EGFP (bottom line).** Representative fluorescent images of fixed MDCK cells after a 1-min (**A, B**) and 5-min (**C, D**) incubation with E69A-mCherry at 1 µM concentration, or after 5-min incubation with OlyA6-mCherry at 2 µM concentration (**E, F**). Nuclei are stained with DAPI (blue). Scale bars: 50 µm.
